# Impact of age and sex correction on the diagnostic performance of dopamine transporter SPECT

**DOI:** 10.1007/s00259-020-05085-2

**Published:** 2020-10-31

**Authors:** Helen Schmitz-Steinkrüger, Catharina Lange, Ivayla Apostolova, Franziska L. Mathies, Lars Frings, Susanne Klutmann, Sabine Hellwig, Philipp T. Meyer, Ralph Buchert

**Affiliations:** 1 Department for Diagnostic and Interventional Radiology and Nuclear Medicine, University Hospital Hamburg-Eppendorf, Martinistr. 52, 20246 Hamburg, Germany; 2 Department of Nuclear Medicine, Berlin Institute of Health, Charité - Universitätsmedizin Berlin, Freie Universität Berlin, Humboldt-Universität zu Berlin, Berlin, Germany; 3 Department of Nuclear Medicine, Medical Center - University of Freiburg, Faculty of Medicine, University of Freiburg, Freiburg, Germany; 4 Department of Psychiatry and Psychotherapy, Medical Center - University of Freiburg, Faculty of Medicine, University of Freiburg, Freiburg, Germany

**Keywords:** Dopamine transporter, SPECT, Specific binding ratio, Age, Sex, Gender

## Abstract

**Purpose:**

The specific binding ratio (SBR) of ^123^I-FP-CIT (FP-CIT) in the putamen decreases with age by about 5% per decade and most likely is about 10% higher in females. However, the clinical utility of age and sex correction of the SBR is still a matter of debate. This study tested the impact of age and sex correction on the diagnostic performance of the putamen SBR in three independent patient samples.

**Methods:**

Research sample: 207 healthy controls (HC) and 438 Parkinson’s disease (PD) patients. Clinical sample A: 183 patients with neurodegenerative parkinsonian syndrome (PS) and 183 patients with non-neurodegenerative PS from one site. Clinical sample B: 84 patients with neurodegenerative PS and 38 patients with non-neurodegenerative PS from another site. Correction for age and sex of the putamen SBR was based on linear regression in the HC or non-neurodegenerative PS, separately in each sample. The area under the ROC curve (AUC) was used as performance measure.

**Results:**

The putamen SBR was higher in females compared to males (PPMI: 14%, *p* < 0.0005; clinical sample A: 7%, *p* < 0.0005; clinical sample B: 6%, *p* = 0.361). Age-related decline of the putamen SBR ranged between 3.3 and 10.4% (*p* ≤ 0.019). In subjects ≥ 50 years, age and sex explained < 10% of SBR between-subjects variance. Correction of the putamen SBR for age and sex resulted in slightly decreased AUC in the PPMI sample (0.9955 versus 0.9969, *p* = 0.025) and in clinical sample A (0.9448 versus 0.9519, *p* = 0.057). There was a small, non-significant AUC increase in clinical sample B (0.9828 versus 0.9743, *p* = 0.232).

**Conclusion:**

These findings do not support age and sex correction of the putaminal FP-CIT SBR in the diagnostic workup of parkinsonian syndromes. This most likely is explained by the fact that the proportion of between-subjects variance caused by age and sex is considerably below the symptom threshold of about 50% reduction in neurodegenerative PS.

**Supplementary Information:**

The online version contains supplementary material available at 10.1007/s00259-020-05085-2.

## Introduction

Parkinson’s disease is characterized by the degeneration of pigmented cells in the substantia nigra pars compacta that results in loss of dopaminergic innervation of the striatum, particularly of the putamen [1, 2]. This is the rationale for the use of SPECT with N-ω-fluoropropyl-2β-carbomethoxy-3β-(4-^123^I-iodophenyl)nortropane (FP-CIT) to assess striatal dopamine transporter (DAT) availability as a marker of nigrostriatal degeneration to support the diagnostic workup of clinically uncertain parkinsonian syndromes (PS) [3–6]. Visual reading of the FP-CIT SPECT images is often complemented by semi-quantitative analysis of striatal DAT availability using the specific binding ratio (SBR) of FP-CIT in the striatum and striatal subregions [5, 7–11].

Post-mortem studies demonstrated that healthy aging is associated with a loss of dopaminergic cells in the substantia nigra and loss of dopamine transporters in the striatum at a rate of about 5% per decade [12–15]. Quantitative and semi-quantitative measures of different components of the dopaminergic system (receptors, transporters) estimated by SPECT and PET in vivo show an age-related decline at about the same rate [16]. In particular, FP-CIT SPECT studies in healthy volunteers quite consistently reported an age-related decline of the putamen SBR ranging between 2.5 and 9.6% per decade (Table 1), in good agreement with the post-mortem studies.

Reported findings on sex differences of striatal DAT availability are less consistent. Although most studies reported higher DAT availability in females compared to males, a considerable proportion of the studies did not find a sex effect on striatal DAT availability, and at least one study reported DAT availability to be lower in females than in males (Table 1).

**Table 1:** Impact of age and sex on semi-quantitative measures of DAT availability in healthy volunteers as measured by SPECT with ^123^I-FP-CIT. Some of the listed studies included the same healthy volunteers from the Parkinson’s Progression Markers Initiative (PPMI) [17], the European Multicentre Database of Healthy Controls for ^123^I-FP-CIT SPECT (ENC-DAT) [18], or from the Japanese Multicentre Database of Healthy Controls for ^123^I-FP-CIT SPECT (JNC-DAT) [19]. Reviews and meta-analyses [16, 20] were not included (occ = occipital, PVE = partial volume effect, FDR = false discovery rate)

Reference	Number of subjects	Age range	% females	Semi-quantitative parameter of DAT availability in the putamen (reference region)	Square of Pearson’s correlation with age (*R*^2^ in %)	Age-related decline (% per decade)	*p* value of age effect	Female-to-male ratio of the semi-quantitative parameter	*p* value of sex effect
[21]	30	41–82	70	SBR (occ)	> 16.8	Anterior putamen: 7.3 Posterior putamen: 6.2	< 0.03		
[22]	133^a^	41–80	50	SBR (occ)	15.2	Striatum: 6.6	< 0.001	n.s.	0.86
[19]	256 (JNC-DAT)	30–83	55	Striatum SBR (Southampton method)	29.1	Women: 7.5 Men: 5.3	< 0.001	30–39 years: 1.12 70–79 years: n.s.	< 0.001
[23]	182 (PPMI)	≥ 30		SBR (cerebellum)	Striatum: 5.1		0.002	n.s	0.24
[24]	181 (PPMI)	≥ 30		SBR (cerebellum)	Spearman *ρ* = − 0.229		0.002		
[25]	30	50–86	43	Striatum SBR (Southampton method)	Women: 25.2 Men: 32.0	8.9		1.13 (estimated from Fig. 3 in [25])	0.036
[26]	73 (ENC-DAT)	20–82	47	Striatum SBR (Southampton method, whole brain w/o striatum)	22.5–33.9	4.1–4.8	< 0.0005		
[27]	182^a^	40–93	60	SBR (occ)		7.0	< 0.001	> 1	0.63
[28]	230^b^	21–85	51	Posterior putamen SBR (occ)	Left: 9.6 Right: 14.4	3.6–4.6	< 0.001	Left: 1.05 Right: 1.06	Left: 0.11 Right: 0.01
[29]	30^c^	48–83 (estimated from figure 3 in [29])	53	Putamen-to-occipital ratio	0.2	2.5	n.s.	> 1	n.s.
[29]	21^d^	74.6 ± 6.3 (at 5 years follow-up)	48	Putamen-to-occipital ratio		Varied between decrease of 5% and increase of 7% between subjects	n.s.	0.85	< 0.05
[30]	122 (ENC-DAT)	20–83	44	PVE-corrected SBR (occ)		Men: 5.6 Females: 5.3	0.0000	1.06	0.031
[31]	123 (ENC-DAT)	20–83	46	SBR (occ)		> 0	0.007	> 1	0.033
[18]	139 (ENC-DAT)	20–83	47	SBR^e^	Women: 42.2 Men: 22.1	Women: 5.9 Men: 4.4	< 0.001	1.09	< 0.009
[32]	51	21–79	65	Voxel-wise SBR (occ)		Left: 4.8 Right: 4.2	FDR-corrected voxel-wise *p* < 0.05	Left: 1.14 Right: 1.20	FDR-corrected voxel-wise *p* < 0.05
[33]	10	40–74	50	Putamen-to-occipital ratio		4.6	0.04	n.s.	0.08
[34]	36	24–83	56	SBR (occ)	19.4	5.0	0.01		
[35]	45	18–83	49	SBR (occ)		4.1	< 0.001	1.1 (estimated from Fig. 1 in [35])	0.005
[37]	14	28–83	50	SBR (occ)	65.6	Striatum: 9.6	< 0.05		

^a^Patients with clinically uncertain parkinsonian syndrome and visually normal FP-CIT SPECT

^b^“patients with normal scans in combination with no evidence of Parkinson’s disease or other neurodegenerative parkinsonism syndromes” [28]

^c^Baseline data

^d^Subjects with FP-CIT SPECT at baseline and after 5 years

^e^With attenuation correction and camera-specific calibration, no scatter correction

Strong evidence of age-related decline of striatal DAT availability and moderate evidence of higher striatal DAT availability in females than in males suggest that correction for age and/or sex might improve the diagnostic performance of semi-quantitative FP-CIT SPECT parameters. However, no studies have yet been published that either clearly confirm or clearly refute this. Albert and co-workers performed a retrospective study to test whether the diagnostic accuracy of the striatal FP-CIT SBR in patients with clinically uncertain PS and previously inconclusive findings in FP-CIT SPECT can be improved by correction for patient age and gamma camera-specific effects [38]. In five patients with inconclusive SPECT according to visual inspection, categorization (as neurodegenerative or non-neurodegenerative) based on age-corrected SBR was correct in three patients (1 true positive, 2 true negative) and false negative in the remaining two patients with clinical follow-up as reference [38].

Thus, there is no clear evidence of a beneficial impact of age and/or sex correction in FP-CIT SPECT in clinical routine. The aim of this study, therefore, was to test the impact of age and sex correction on the diagnostic performance of the putaminal FP-CIT SBR in three independent datasets comprising a total of 1133 subjects.

## Materials and methods

### PPMI sample

Baseline FP-CIT images of 645 subjects, 207 healthy control (HC) subjects and 438 patients with Parkinson’s disease (PD), were obtained from the Parkinson’s Progression Markers Initiative (PPMI) (www.ppmi-info.org/data) [39]. Up-to-date information on the PPMI is given at www.ppmi-info.org. The PPMI is a longitudinal, multi-center study that aims to assess the progression of clinical features, imaging, and biologic markers in patients with PD and in HC subjects. Details of the PPMI eligibility criteria and of the PPMI FP-CIT SPECT protocol are given at http://www.ppmi-info.org/wp-content/uploads/2014/01/PPMI-AM7-Protocol.pdf and http://www.ppmi-info.org/study-design/research-documents-and-sops/, respectively [39]. SPECT images were reconstructed by the PPMI imaging core lab using an iterative ordered-subsets-expectation-maximization algorithm with eight iterations and eight subsets and no filtering [40]. Post-reconstruction attenuation correction according to Chang was performed using a site-specific attenuation coefficient followed by 3-dimensional Gaussian filtering with 6 mm full-width-at-half-maximum [17]. Correction for photon scatter was not performed [40].

Confirmation from the PPMI imaging core lab that the FP-CIT SPECT was consistent with DAT deficit was a major inclusion criterion for PD patients in the PPMI [41]. Subjects screened as potential PD patients who were not eligible due to DAT imaging without evidence of dopaminergic deficit (SWEDD) were enrolled in a separate PPMI SWEDD cohort [41]. The present study did not include PPMI SWEDD subjects.

### Clinical sample A

Three-hundred-and-sixty-six patients from clinical routine were recruited retrospectively from the database of the University Medical Center Hamburg-Eppendorf. Data of these patients have been used previously to test the impact of the size of the normal database on the performance of the putamen SBR in FP-CIT SPECT [42].

The patients were categorized into “neurodegenerative PS” and “non-neurodegenerative PS”. The neurodegenerative group (*n* = 183) comprised 150 patients (82%) with PD, 27 patients (15%) with atypical neurodegenerative PS (multiple systems atrophy, progressive supranuclear palsy, corticobasal degeneration), and 6 patients (3%) with dementia with Lewy bodies. The non-neurodegenerative group (*n* = 183) comprised essential tremor, drug-induced parkinsonism, several types of dystonia, psychogenic parkinsonism, and various other diagnoses not associated with relevant nigrostriatal degeneration. The clinical diagnoses as standard of truth were taken from the report of a movement disorder specialist in the patient’s file written at least 12 months after FP-CIT SPECT in all 183 patients with neurodegenerative PS (mean follow-up 41 ± 22 months, range 12–95 months) and in 44 of the patients with non-neurodegenerative PS (mean follow-up 38 ± 22 months, 13–97 months). The remaining patients with non-neurodegenerative PS had less than 12 months follow-up and were included to increase sample size and to avoid imbalance with respect to the diagnostic class (neurodegenerative versus non-neurodegenerative).

FP-CIT SPECT projection data had been acquired with a double-head camera (Siemens Symbia T2) equipped with fan-beam collimators in 214 (58.5%) patients (60 projections of 40 s duration with each head along a scan arc of 180°, 128 × 128 matrix, zoom 1.23). A double-head camera (Siemens E.CAM) equipped with low-energy high-resolution parallel-hole collimators had been used in the remaining 152 (41.5%) patients (64 projections of 35 s duration with each head along a scan arc of 180°, 128 × 128 matrix, zoom 1.0). The energy window was 147–171 keV, injected dose of FP-CIT was 196 ± 21 MBq, and radius of rotation was 16.1 ± 1.8 cm. In order to ensure consistent image reconstruction in all patients, projection data were retrieved from the archive and reconstructed retrospectively according to the standard operating procedure of the University Medical Center Hamburg-Eppendorf for FP-CIT SPECT (filtered backprojection implemented in the SPECT system software, Butterworth filter of 5th order with cutoff 0.6 cycles/pixel, uniform post-reconstruction attenuation correction according to Chang with *μ* = 0.12/cm, no scatter correction).

### Clinical sample B

One-hundred-and-twenty-two patients from clinical routine were recruited retrospectively from the database of the Department of Nuclear Medicine of the University Medical Center Freiburg. Data of these patients have been used previously to test the diagnostic performance of the specific uptake size index for semi-quantitative analysis of FP-CIT SPECT [43].

The patients were retrospectively categorized into “neurodegenerative PS” and “non-neurodegenerative PS”. The neurodegenerative group (*n* = 84) comprised 46 patients (55%) with PD, 24 patients (28%) with atypical neurodegenerative PS, and 14 patients (17%) with dementia with Lewy bodies. The non-neurodegenerative group (*n* = 38) comprised essential tremor, vascular parkinsonism, drug-induced parkinsonism, psychogenic parkinsonism, possible Alzheimer’s disease, and normal pressure hydrocephalus. The clinical diagnoses as standard of truth were established retrospectively by a movement disorder specialist in accordance with consensus criteria [44] based on systematic review of all relevant medical charts and clinical follow-up data (mean follow-up 26.8 ± 14.5 months) [45].

The same double-head SPECT system (Siemens E.CAM) equipped with low-energy high-resolution parallel-hole collimators had been used in all patients (60 projections of 30 s duration with each head along a scan arc of 180°, 128 × 128 matrix, zoom 1.23). The energy window was 144–168 keV, injected dose of FP-CIT was 193 ± 8 MBq, and radius of rotation was 13.5 ± 0.3 cm. In order to ensure consistent image reconstruction in all patients, projection data were exported from the archive and retrospectively reconstructed according to the standard operating procedure of the University Medical Center Freiburg for FP-CIT SPECT [46] (3-dimensional ordered-subset-expectation-maximization with resolution recovery using the Flash3D algorithm of the scanner software, uniform post-reconstruction attenuation correction according to Chang with *μ* = 0.12/cm, no scatter correction).

The neurodegenerative subgroups of the clinical samples were representative of the spectrum of patients with neurodegenerative PS referred to FP-CIT SPECT at the clinical sites. In particular, the neurodegenerative subgroups of the clinical samples included patients with atypical neurodegenerative PS and dementia with Lewy bodies, in contrast to the neurodegenerative subgroup of the PPMI sample that included only PD patients.

### Semi-quantitative SBR analysis

Individual SPECT images were transformed (affine) into the anatomical space of the Montreal Neurological Institute (MNI) using the Statistical Parametric Mapping software package (version SPM12) [47] and a custom-made FP-CIT template. Voxel intensities were scaled to the 75th percentile in a reference region comprising whole brain except striata, thalamus, brain stem, and ventricles [48, 49].

Hottest voxels analysis in large unilateral ROIs predefined in MNI space was used to compute the unilateral putamen SBR [43]. More precisely, the unilateral putamen SBR was calculated as mean scaled voxel intensity in the 10-ml hottest ROI voxels − 1. The minimum of the unilateral putamen SBR of left and right hemisphere was used in all analyses.

All analyses were performed at one site using the same custom-made MATLAB/SPM12 script for fully automatic processing (including stereotactical normalization, intensity scaling, and semi-quantitative analysis) in batch mode in order to avoid operating errors by manual analysis.

### Statistical analysis

Age was compared between two groups using the homoscedastic or the heteroscedastic two-sample *t* test depending on the result of Levene’s test of the homogeneity of the variances. The sex distribution was compared between two groups using Pearson’s chi-square test.

Linear regression of the putamen SBR was performed with age and sex (female = 0, male = 1) as independent variables. This was performed separately in the HC subjects of the PPMI sample, in the patients with a non-neurodegenerative PS of clinical sample A, and in the patients with a non-neurodegenerative PS of clinical sample B. The regression lines were used to estimate the mean relative age-related decline of the putamen SBR in percent per decade and the difference of the putamen SBR between females and males. Age 30 years was used as reference.

The resulting regression lines were also used to correct the SBR for age and sex in all subjects, separately in each sample. The performance of the SBR with and without age and sex correction for identification of PD (PPMI sample) or for identification of a neurodegenerative PS (clinical samples) was assessed using receiver operating characteristic (ROC) analysis. The DeLong method was used to test the impact of age and sex correction on the area under the ROC curve for statistical significance [50]. In each sample, ROC analysis first included all subjects. In addition, ROC analysis was restricted to the youngest 25% of subjects (lower quartile) or to the oldest 25% of subjects (upper quartile) in the sample.

Constant cutoffs (independent of age and sex) for SBR-based classification of FP-CIT SPECT as “normal” or “reduced” were determined from the ROC curves by maximization of the Youden index *J* = sensitivity + specificity − 1 [51]. In order to test for potential improvement of SBR-based classification by using an age-dependent cutoff, a linear cutoff = cutoff_0_ − ß*age was fitted by optimizing the Youden index. A grid search was used for this purpose to avoid trapping in local minima. The search grid was determined based on linear regression of the putamen SBR with age as independent variable in the HC subjects (PPMI sample) or in the patients with non-neurodegenerative PS (clinical samples): SBR = SBR_0_ − μ*age. More precisely, the search grid covered cutoff_0_ ranging from 0 to SBR_0_ and ß ranging from 0 to 2*μ.

All statistical analyses were performed with SPSS statistics (version 25, IBM) except the DeLong test that was performed with the R software package. An effect was considered statistically significant if the two-sided *p* was below 0.05.

Additional analyses are given in the electronic supplementary material, including (i) testing the impact of age and sex correction on the diagnostic performance of the caudate nucleus SBR and of the putamen-to-caudate SBR ratio, (ii) testing the impact of the reconstruction method on the effect of age and sex on the putamen SBR, and (iii) testing the impact of the method used to estimate the SBR on age and sex effects.

## Results

**Fig. 1:**
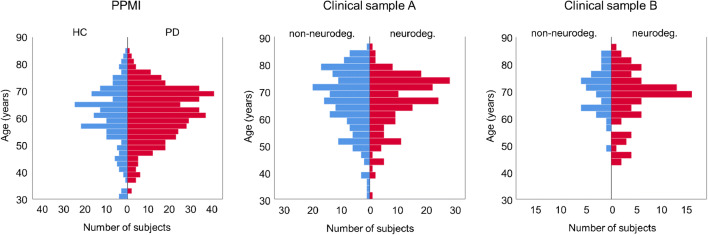
Age distribution in the different samples

Neither age nor sex differed significantly between healthy controls and patients with PD in the PPMI sample nor between patients with non-neurodegenerative PS and patients with neurodegenerative PS in any of the two clinical samples (Table 2, Fig. 1).

**Table 2:** Demographics

	PPMI sample			Clinical sample A			Clinical sample B		
	HC	PD	p	Non-neurodeg.	Neurodeg.	*p*	Non-neurodeg.	Neurodeg.	*p*
*n*	207	438		183	183		38	84	
Age, mean ± standard deviation (range)	60.5 ± 11.2 (30.8–84.2)	61.5 ± 9.7 (33.6–84.8)	0.232	66.1 ± 11.6 (30.9–86.0)	66.1 ± 10.1 (31.4–85.1)	0.993	70.0 ± 8.0 (48.0–84.5)	67.3 ± 10.0 (44.1–86.1)	0.141
% females	35.3	35.2	0.979	51.9	45.9	0.250	39.5	46.4	0.474

**Fig. 2:**
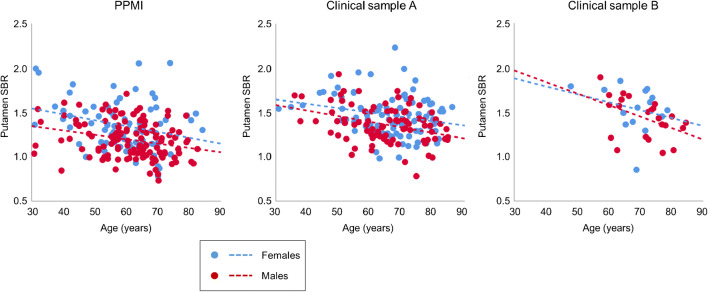
Scatter plot of the (uncorrected) putamen SBR versus age in the HC subjects (PPMI sample) and in the patients with non-neurodegenerative PS (clinical samples). Sex is indicated by different colors. The dashed lines represent the result of linear regression of the putamen SBR versus age, performed separately for both sexes

Mean putamen SBR in the different samples is given in Table 3. Linear regression of the putamen SBR with age and sex as independent variables in the healthy controls (PPMI sample) or the patients with non-neurodegenerative PS (clinical samples) resulted in the following regression lines (Fig. 2):

$$ \mathrm{PPMI}\ \mathrm{sample}\ \left(\mathrm{HC}\right):\mathrm{SBR}=1.342\hbox{--} 0.558\ast \mathrm{age}/100\hbox{--} 0.148\ast \mathrm{sex} $$

clinical sample A (non − neurodeg. PS) : SBR = 1.838 – 0.559 ∗ age/100 – 0.113 ∗ sex

$$ \mathrm{clinical}\ \mathrm{sample}\ \mathrm{B}\ \left(\mathrm{non}-\mathrm{neurodeg}.\mathrm{PS}\right):\mathrm{SBR}=1.518\hbox{--} 1.151\ast \mathrm{age}/100\hbox{--} 0.070\ast \mathrm{sex} $$

**Table 3:** Specific FP-CIT binding ratio (SBR) of the putamen (minimum of both hemispheres) without and with correction for age and sex (mean ± standard deviation)

	PPMI sample				Clinical sample A				Clinical sample B			
	HC		PD		Non-neurodeg.		Neurodeg.		Non-neurodeg.		Neurodeg.	
	Females	Males	Females	Males	Females	Males	Females	Males	Females	Males	Females	Males
*n*	73	134	154	284	95	88	84	99	15	23	39	45
Age (years)	59.1 ± 11.8	61.2 ± 10.9	60.7 ± 9.7	62.0 ± 9.7	66.9 ± 11.4	65.4 ± 11.8	66.6 ± 9.8	65.7 ± 10.4	68.3 ± 7.7	71.1 ± 8.1	68.0 ± 10.4	66.6 ± 9.7
Uncorrected putamen SBR	1.35 ± 0.28	1.19 ± 0.20	0.50 ± 0.15	0.47 ± 0.16	1.46 ± 0.23	1.36 ± 0.21	0.66 ± 0.32	0.69 ± 0.33	1.54 ± 0.26	1.43 ± 0.23	0.66 ± 0.24	0.70 ± 0.28
Age and sex corrected putamen SBR	1.34 ± 0.27	1.34 ± 0.19	0.50 ± 0.16	0.63 ± 0.17	1.84 ± 0.22	1.84 ± 0.19	1.03 ± 0.33	1.17 ± 0.34	1.52 ± 0.25	1.52 ± 0.20	0.64 ± 0.22	0.73 ± 0.30

Thus, the regression coefficients were negative for both age and sex, indicating age-related decline and higher SBR in females compared to males in all samples. The SBR was higher in females compared to males by 14%, 7%, and 6% in the PPMI sample, clinical sample A, and clinical sample B, respectively. Mean relative age-related decline of the putamen SBR per decade in females/males was 4.8%/5.4% in the PPMI sample, 3.3%/3.6% in clinical sample A, and 9.8%/10.4% in clinical sample B. The effects of age and sex were highly significant in the PPMI sample and in clinical sample A (Table 4). In clinical sample B, only age reached statistical significance (Table 4).

**Table 4:** Results of the linear regression of the putamen SBR with age and sex as independent variables in healthy controls (PPMI sample) and patients with non-neurodegenerative PS (clinical samples)

	Healthy controls of the PPMI sample (total *n* = 207, 73 females, 134 males)	Patients with non-neurodegenerative PS of clinical sample A (total *n* = 183, 95 females, 88 males)	Patients with non-neurodegenerative PS of clinical sample B (total *n* = 38, 15 females, 23 males)
Age-related decline of putamen SBR (% per decade) in females/males	4.8/5.4	3.3/3.6	9.8/10.4
Standardized coefficient *β* of sex	− 0.293 (*p* < 0.0005)	− 0.255 (*p* < 0.0005)	− 0.144 (*p* = 0.361)
Standardized coefficient *β* of age	− 0.258 (*p* < 0.0005)	− 0.290 (*p* < 0.0005)	− 0.381 (*p* = 0.019)
Total between-subjects variance explained (adjusted *R*^2^ in %)	15.8	13.0	13.8
Total between-subjects variance explained (adjusted *R*^2^ in %) in subjects 50 years and older	7.9	7.5	9.4

The total between-subjects variance explained by the regression model (adjusted *R*^2^ [52]) was 15.8%, 13.0%, and 13.8% in the PPMI sample, in clinical sample A, and in clinical sample B, respectively. When the regression was restricted to subjects of 50 years and older, the between-subjects variance explained by the regression dropped to 7.9%, 7.5%, and 9.4% in the PPMI sample, in clinical sample A, and in clinical sample B, respectively. For comparison, total between-subjects variance of the putamen SBR in all subjects of 50 years and older explained by the group (PD versus HC in the PPMI sample, neurodegenerative versus non-neurodegenerative PS in the clinical samples) was 77.8%, 61.9%, and 71.6% in the PPMI sample, clinical sample A, and clinical sample B, respectively.

Linear regression of the putamen SBR with age and sex as independent variables in the PD patients (PPMI sample) or in the patients with neurodegenerative PS (clinical samples) did not reveal a significant effect (PPMI sample: standardized coefficient *β* of age = 0.044, *p* = 0.362; *β* of sex = −0.085, *p* = 0.076; clinical sample A: *β* of age = 0.130, *p* = 0.081; *β* of sex = 0.041, *p* = 0.578; clinical sample B: *β* of age = − 0.157, *p* = 0.156; *β* of sex = 0.064, *p* = 0.561).

The regression lines were used to correct the SBR for age and sex in all subjects, separately in each sample, using the following formulas:

$$ \mathrm{PPMI}\ \mathrm{sample}\ \left(\mathrm{all}\ \mathrm{subjects}\right):\mathrm{corrected}\ \mathrm{SBR}=\mathrm{uncorrected}\ \mathrm{SBR}+0.558\ast \mathrm{age}/100+0.148\ast \mathrm{sex} $$

clinical sample A (all patients) : corrected SBR = uncorrected SBR + 0.559 ∗ age/100 + 0.113 ∗ sex

$$ \mathrm{clinical}\ \mathrm{sample}\ \mathrm{B}\ \left(\mathrm{all}\ \mathrm{patients}\right):\mathrm{corrected}\ \mathrm{SBR}=\mathrm{uncorrected}\ \mathrm{SBR}+1.151\ast \mathrm{age}/100+0.070\ast \mathrm{sex} $$

**Fig. 3:**
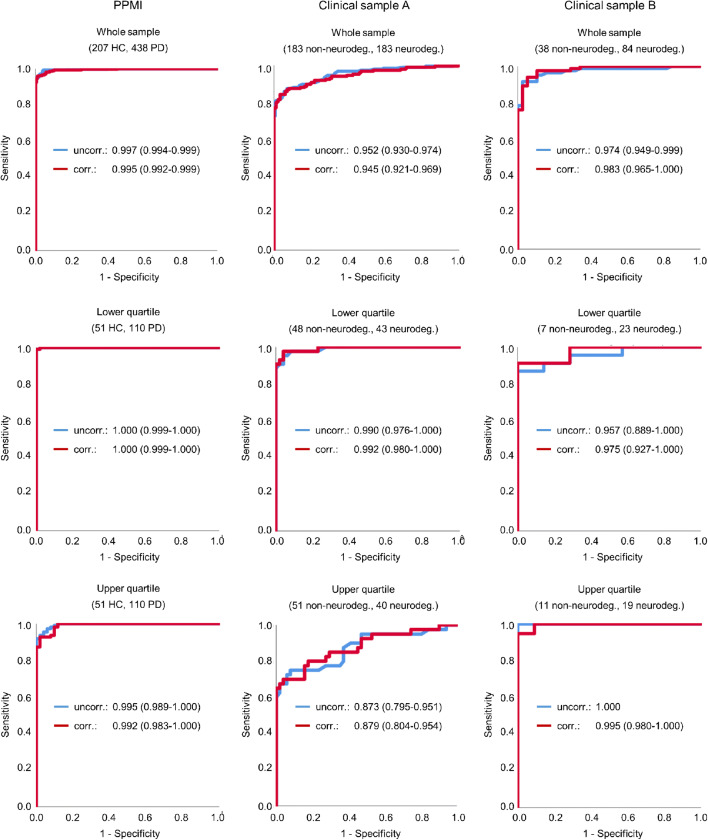
ROC curves of the putamen SBR with and without correction for age and sex in the different samples. The ROC curves in the top row include all subjects of the corresponding sample. The ROC curves in the middle and in the bottom row include only “young” subjects (lower quartile with respect to age in the corresponding sample) or only “old” subjects (upper quartile). The area under the ROC curve and its 95%-confidence interval is given in the legends to the ROC curves

Mean corrected putamen SBR in the different samples is given in Table 3. The results of the ROC analyses are summarized in Table 5 and Fig. 3. The only statistically significant effect was a slight decrease of the area under the ROC curve by age and sex correction in the whole PPMI sample from 0.9969 without correction to 0.9955 with age and sex correction (*p* = 0.025). The slight decrease of the area under the ROC curve by age and sex correction in the whole clinical sample A from 0.9519 to 0.9448 reached trend level significance (*p* = 0.057). In clinical sample B, age and sex correction resulted in a small increase of the area under the ROC curve from 0.9743 to 0.9828, which however was not statistically significant (*p* = 0.232).

**Table 5:** Results of the ROC analyses of the putamen SBR to detect PD (PPMI sample) or a neurodegenerative PS (clinical samples) (*AUC*, area under the ROC curve; *VAR*, variance of the AUC; *SE*, standard error of the AUC; 95%-CI, 95%-confidence interval)

		Uncorrected SBR				Corrected SBR				
	Age group Mean age (years) ± standard deviation (range)	AUC	VAR	SE	95%-CI	AUC	VAR	SE	95%-CI	DeLong *p*
PPMI	Whole sample 61.2 ± 10.2 (30.8–84.8)	0.9969	1.61E−06	0.0013	0.9944–0.9994	0.9955	2.53E−06	0.0016	0.9924–0.9986	0.0246
	Youngest quartile 47.5 ± 6.2 (30.8–54.6)	0.9998	6.35E−08	0.0003	0.9993–1	0.9998	6.35E−08	0.0003	0.9993–1	1
	Oldest quartile 73.3 ± 3.8 (69.0–84.8)	0.9952	8.17E−06	0.0029	0.9896–1	0.9916	1.93E−05	0.0044	0.9830–1	0.1356
Clinical sample A	Whole sample 66.1 ± 10.8 (30.9–86.0)	0.9519	1.23E−04	0.0111	0.9302–0.9736	0.9448	1.53E−04	0.0124	0.9206–0.9690	0.0569
	Youngest quartile 50.9 ± 6.6 (30.9–59.5)	0.9903	4.73E−05	0.0069	0.9768–1	0.9922	3.67E−05	0.0061	0.9804–1	0.2867
	Oldest quartile 78.2 ± 3.0 (74.3–86.0)	0.8730	1.60E−03	0.0400	0.7946–0.9515	0.8787	1.48E−03	0.0385	0.8032–0.9542	0.6546
Clinical sample B	Whole sample 68.1 ± 9.5 (44.1–86.1)	0.9743	1.63E−04	0.0128	0.9493–0.9993	0.9828	8.12E−05	0.0090	0.9651–1	0.2319
	Youngest quartile 54.9 ± 6.4 (44.1–63.3)	0.9565	1.12E−03	0.0335	0.8909–1	0.9752	5.52E−04	0.0235	0.9291–1	0.2506
	Oldest quartile 78.9 ± 3.1 (74.3–86.1)	1	0	0.0000	1–1	0.9952	4.58E−05	0.0068	0.9820–1	0.4795

**Fig. 4:**
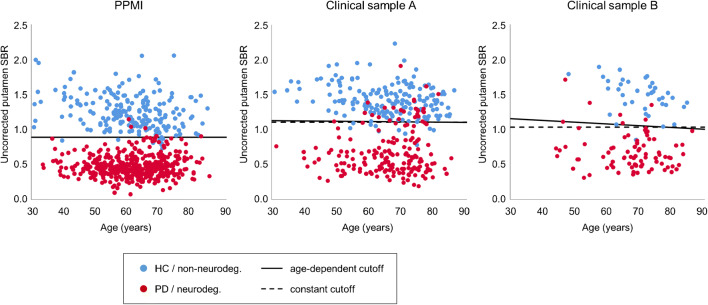
Scatter plot of the (uncorrected) putamen SBR versus age in the different samples. The solid line shows the age-dependent cutoff optimized for detection of PD (PPMI sample) or neurodegenerative PS (clinical samples) with maximum balanced accuracy. The dashed lines represent the constant cutoff with maximum balanced accuracy. In the PPMI sample, balanced accuracy could not be improved by allowing the cutoff to decline with age (solid line and dashed line coincide)

Sensitivity and specificity of the uncorrected putamen SBR for detection of a neurodegenerative PS using the constant cutoff (independent of age) were 98.9% and 96.1% in the PPMI sample, 85.8% and 94.5% in clinical sample A, and 91.6% and 97.4% in clinical sample B (Fig. 4). Classification based on the age-dependent cutoff agreed with classification based on the constant cutoff in 1131 of the 1133 subjects (99.8%). In clinical sample A, the classification of a 49-year-old male diagnosed with PD after clinical follow-up of 30 months was corrected to neurodegenerative PS when using the age-dependent cutoff (0.4% decline per decade). In clinical sample B, the classification of a 46-year-old female diagnosed with PD after clinical follow-up of 37 months was corrected to neurodegenerative PS when using the age-dependent cutoff (2.2% decline per decade). This resulted in increased sensitivity in the clinical samples by the age-dependent cutoff (from 85.8 to 86.3% in clinical sample A, from 91.6 to 92.8% in clinical sample B). Specificity of the uncorrected putamen SBR in the clinical samples was not changed by the age-dependent cutoff. In the PPMI sample, neither sensitivity nor specificity could be improved by allowing the cutoff to decline with age (Fig. 4).

## Discussion

The primary finding of this study was that correction for age and sex did not significantly improve the performance of the putamen SBR for the detection of neurodegenerative parkinsonian syndromes in any of the three samples, neither in the whole samples nor in the youngest or in the oldest quartiles. Minor deterioration of the diagnostic performance of the putamen SBR by age and sex correction in the whole PPMI sample and in the whole clinical sample A most likely is explained by the higher fraction of subjects with clinical diagnosis of neurodegenerative PS and normal FP-CIT SPECT (SWEDD) at older age (Fig. 4).

The primary finding was confirmed by the fact that allowing the cutoff on the uncorrected putamen SBR to decline with age resulted in correction of the classification (according to the constant cutoff) in only 2 of the 1133 subjects included in this study. This small improvement of diagnostic accuracy might be explained by stronger overfitting of the age-dependent cutoff compared to the constant cutoff (one additional free fit parameter), because diagnostic performance was estimated in the same patient sample that was used for optimization of the cutoff. In contrast, the explicit age and sex correction of the putamen SBR based on linear regression in the HC subjects (PPMI sample) or in the patients with a non-neurodegenerative PS (clinical samples) did not use information from the PD patients (PPMI sample) or the patients with neurodegenerative PS (clinical samples) and, therefore, was less prone to overfitting. This most likely explains the minor differences of the results obtained with these two different correction methods (explicit age and sex correction of the putamen SBR versus age-dependent cutoff).

Additional analyses reported in the electronic supplementary material suggested that the lack of relevant improvement of the diagnostic accuracy of the putamen SBR by age and sex correction can be generalized to other reconstruction algorithms and to other methods to estimate the putamen SBR.

The lack of a clear benefit from age and sex correction of the putamen SBR in the present study is in line with a previous study that reported age not to affect sensitivity and specificity of visual interpretation of FP-CIT SPECT [53]. The authors of a study to compare FP-CIT SPECT and F-DOPA PET in patients with PD and healthy controls concluded that “the aging effect on striatal DAT binding (of FP-CIT) is relatively small and adjusting uptake values for age did not influence its accuracy for diagnosing presynaptic dopaminergic deficit” but did not report the underlying results [33]. A study to assess the accuracy and reproducibility of a software tool (BasGan) for computer-assisted analysis of FP-CIT SPECT reported the area under the ROC curve of the SBR in the more severely affected putamen for detection of a neurodegenerative PS to be slightly smaller with correction for age than without correction for age (0.898 versus 0.909), although the difference was not statistically significant [54]. Age correction had been performed by computing the ratio of the patient’s SBR to the SBR of normal subjects of similar age in this previous study [54].

The main reason for the lack of a beneficial effect of age and sex correction on the classification performance of the putamen SBR in patients with a PS most likely is the rather low proportion of between-subjects variance that is explained by age and sex relative to the rather high symptom threshold. Post-mortem studies have shown that motor symptoms in PD start at rather advanced stages of nigrostriatal degeneration when loss of dopamine neurons has reached about 50% [12], depletion of striatal dopamine has reached about 70% [1], and loss of DAT in the unilateral posterior putamen has reached about 50% [55]. These post-mortem findings where supported by an FP-CIT SPECT study that estimated the symptom threshold to lie between 46 and 64% reduction of the putamen SBR (from its value in young adulthood), independent of age [34]. Thus, the symptom threshold is about three to four times larger than the proportion of between-subjects variance of the putaminal SBR that is explained by age and sex: 15.8% in the PPMI sample, 13.0% in clinical sample A, 13.8% in clinical sample B (Fig. 2).

It should be noted in this context that most studies on aging of the dopaminergic system were cross-sectional and covered a very wide age range (typically about 20 to about 80 years; Table 1) rather than focusing on older adults representing the typical age range of patients referred to FP-CIT SPECT for suspicion of a neurodegenerative PS. This point is practically relevant, because the proportion of between-subjects variance of the putaminal SBR that is explained by age and sex decreases when the considered age range is reduced from 20–80 years to 50–80 years. In the PPMI sample, for example, the proportion of the between-subjects variance of the putamen SBR explained by age and sex decreased from 15.8% in all healthy PPMI subjects (30–84 years) to 7.9% in the healthy PPMI subjects older or equal 50 years. In line with this, Roberts and co-workers did not find an age-related decline of the striatal SBR in 29 healthy volunteers and 23 patients with mild cognitive impairment due to Alzheimer’s disease (not associated with nigrostriatal degeneration) aged between 60 and 92 years [56]. In a longitudinal study that performed two FP-CIT SPECT with a delay of five years in 21 healthy subjects aged 74.6 ± 6.3 years (at the follow-up SPECT), the individual annual relative change of the putamen-to-occipital FP-CIT uptake ratio varied between 5% decrease and 7% increase [29]. The mean annual relative change did not reach statistical significance (for being different from zero) in this longitudinal study [29].

There are early signs of PD such as smell loss and rapid eye movement sleep disorder that can precede motor symptoms by several years, but are not particularly specific for PD [57–59]. It might become increasingly important to detect PD also at these early stages when the loss of striatal DAT is below the threshold of motor symptoms, because the chance to moderate the course of PD by disease-modifying drugs most likely is better at early disease stages [60]. Age and sex correction of the putamen SBR might be more beneficial at early premotor stages, when the loss of DAT in the putamen is of about the same size (or smaller) than the between-subjects variability of the putamen SBR related to age and sex. However, the diagnostic utility of FP-CIT SPECT at these early disease stages might be limited by test-retest variability. Test-retest variability of 7.8 ± 4.6% has been reported for the putamen SBR [61]. Considerably higher test-retest variability of the SBR of 13.7 ± 9.9 / 12.2 ± 8.5% has been reported for the posterior part of the left/right putamen [62], where PD-related loss of striatal DAT starts [55]. Thus, test-retest variability of the SBR is of about the same size as the between-subjects variability due to age and sex. This further limits the potential of age and sex correction to improve the diagnostic performance of the putamen SBR.

The sensitivity of the putamen SBR for detection of neurodegenerative PS was considerably higher in the PPMI sample (98.9% for uncorrected putamen SBR with constant cutoff) compared to clinical samples A (85.8%) and B (91.6%). This most likely is explained by the exclusion of SWEDD subjects in the PD cohort of the PPMI [41]. A ceiling effect due to the good (clinical samples) to very good (PPMI sample) performance of the uncorrected SBR might also have contributed to the lack of a beneficial impact of age and sex correction in this study (diagnostic accuracy is bounded from above at 100%, which limits the effect size of potential improvement, which in turn limits the statistical power for detection of the improvement).

It should be noted that the relative SBR decline per decade depends on the reference age, at least in case of a linear age decline (implying constant *absolute* annual SBR loss, independent of age). The *relative* annual loss then increases with age, because the reference value decreases with age. Additional variability (of no interest) between studies caused by this effect can be avoided by using the same fixed reference age in all studies (e.g., 30 years as in the present study) or by using another age model (e.g., an exponential model [63]). Some studies use the ROI-to-reference ratio to characterize striatal FP-CIT binding instead of the SBR (= ROI-to-reference ratio − 1; Table 1). As age-related decline differs between the ROI-to-reference ratio and the SBR, it is important to specify which of both is used when reporting age-related decline.

The present study showed 6–14% higher mean putamen SBR in females compared to males, in line with several previous studies (Table 1). The effect was statistically significant in the PPMI sample and in clinical sample A (both *p* < 0.0005), but it clearly missed the significance level in clinical sample B (*p* = 0.361). To some extent this might be explained by lower statistical power due to considerably smaller sample size of clinical sample B (Table 4). The fact that also previous studies were less consistent with respect to the impact of sex on the putamen SBR than with respect to its age-related decline (Table 1) suggests that the sex effect is weaker than the age effect. Furthermore, the sex effect on striatal DAT availability might be most prominent at young age. A previous study reported 12% higher striatal SBR in healthy women compared to men in the 30–39 years age range but no difference in the 70–79 years age group [19]. The present study included only very few subjects in the 30–39 years age range (Fig. 1).

Given that the putamen volume is on average 7–8% smaller in females compared to males [64] and that no correction of partial volume effects was performed in this study, the difference in putamen SBR between females and males most likely was underestimated here. Experimental data suggest that higher striatal DAT density in females compared to males is a genomic effect [36] and that striatal DAT density in females fluctuates during the estrous cycle [65].

Limitations of the present study include the following. First, no correction for partial volume effects was performed. This might have resulted in an overestimation of the age-related decline of putaminal DAT density, as the putamen shrinks during healthy aging with a rate of approximately 3.6% per decade within a span of 20 to 80 years of age [64]. Second, age correction was based on the same healthy control subjects (PPMI sample) or the same patients with non-neurodegenerative PS (clinical samples) that were used in the ROC analyses to assess the impact of age and sex correction on the diagnostic performance of the putamen SBR. However, the potential bias that might have been caused by this more likely resulted in overestimation than in underestimation of the benefit of the age and sex correction. Third, the vast majority of the subjects included in this study was 50 years or older: 86.0%, 91.5%, and 93.4% in the PPMI sample, clinical sample A, and clinical sample B, respectively. Thus, the findings mainly apply to this age group. The impact of age correction on the diagnostic performance of FP-CIT SPECT in juvenile and young-onset parkinsonian syndromes should be further tested in future studies. This will also allow more detailed analysis of sex and sex*age interaction effects on striatal DAT availability, as sex effects might be more pronounced at younger age [19]. Finally, the standard of truth diagnosis of the patients of the clinical samples was established by a movement disorder specialist who was not blinded with respect to the FP-CT SPECT finding. This might have caused some bias in favor of FP-CIT SPECT that resulted in overestimation of the diagnostic performance of the putamen SBR. However, the rather large number of SWEDD subjects in the clinical samples and the fact that the diagnostic accuracy of the putamen SBR in the clinical samples was in good agreement with previous studies in clinically uncertain PS [66] suggest that the bias was not very large. Furthermore, the standard of truth diagnoses of the patients in clinical sample A were taken from the report in the patient’s file written after FP-CIT SPECT (at least 12 months in most patients). The vast majority of the reports did not explicitly state the criteria used for the diagnosis. We therefore can only assume that the diagnoses were based on established criteria.

## Conclusion

The specific putaminal FP-CIT binding ratio decreases during healthy aging at a rate of 3.3–10.4% per decade and is 6–14% higher in females than in males. In patients aged 50 years and older, correction for age and sex does not improve its accuracy in the differentiation of neurodegenerative parkinsonian syndromes from non-neurodegenerative parkinsonian syndromes. This most likely is explained by the fact that age and sex explain less than 10% of the between-subjects variance of the specific putaminal FP-CIT binding ratio in subjects aged 50 years and older, which is considerably below the symptom threshold of about 50% reduction in neurodegenerative PS.

## Supplementary Information

ESM 1 (DOCX 456 kb)

## Data Availability

The datasets supporting the conclusions of this article can be made available on request.

## Abbreviations

AUC: Area under the ROC curve
DAT: Dopamine transporter
FP-CIT: N-ω-fluoropropyl-2β-carbomethoxy-3β-(4-I-123-iodophenyl)nortropane
HC: Healthy control
MNI: Montreal Neurological Institute
PD: Parkinson’s disease
PPMI: Parkinson’s Progression Marker Initiative
PS: Parkinsonian syndrome
ROC: Receiver operating characteristic
SBR: Specific binding ratio
SPECT: Single-photon emission computed tomography
SWEDD: Subject without evidence of dopaminergic deficit
