# Supplementary material for: Impact of age and sex correction on the diagnostic performance of dopamine transporter SPECT
Source: Eur J Nucl Med Mol Imaging. 2020 Oct 31;48(5):1445–59. doi: 10.1007/s00259-020-05085-2 (PMC8113204; doi:10.1007/s00259-020-05085-2)
Supplement: Supplementary file 1 — (DOCX 456 kb) [file 259_2020_5085_MOESM1_ESM.docx]

**Electronic Supplementary Material**

Title: Impact of age and sex correction on the diagnostic performance of dopamine transporter SPECT

Journal: European Journal of Nuclear Medicine and Molecular Imaging

Authors: Helen Schmitz-Steinkrüger, Catharina Lange, Ivayla Apostolova, Franziska L. Mathies, Lars Frings, Susanne Klutmann, Sabine Hellwig, Philipp T. Meyer, Ralph Buchert

Corresponding author: Ralph Buchert, Martinistr. 52, 20246 Hamburg, Germany, Email: [r.buchert@uke.de](mailto:r.buchert@uke.de), Phone: +49 40 7410 54347, Fax: +49 40 7410 40265, ORCID ID 0000-0002-0945-0724

**Specific binding ratio of the caudate nucleus and putamen-to-caudate ratio**

The unilateral specific binding ratio (SBR) of the caudate nucleus in the PPMI sample was computed using hottest voxels analysis in large unilateral ROIs for the caudate nucleus (figure 1C in [1]). More precisely, the unilateral caudate SBR was calculated as mean scaled voxel intensity in the 5 ml hottest voxels in the unilateral caudate ROI – 1. Then, the putamen-to-caudate SBR ratio was computed separately for left and right hemisphere. The minimum of the putamen-to-caudate SBR ratio of left and right hemisphere was used in all further analyses (analogous to the minimum of the unilateral putamen SBR of left and right hemisphere that was used in the analyses in the manuscript).

The ROC curve of the putamen-to-caudate SBR ratio for detection of PD in the PPMI sample is shown in supplementary Figure S1 below, together with the ROC curve of the putamen SBR and the caudate SBR (minimum of both hemispheres). The area under the ROC curve was largest for the putamen SBR (0.997, 95%-confidence interval 0.994-0.999) and smallest for the caudate SBR (0.946, 0.930-0.962). The area under the ROC curve of the putamen-to-caudate SBR ratio was in between (0.986, 0.979-0.993). All pairwise differences of the area under the ROC curve were statistically significant (p ≤ 0.004).

Linear regression of the caudate SBR with age and sex as independent variables in the healthy controls of the PPMI sample revealed a significant effect of age and sex (standardized coefficient β of age = -0.393, p < 0.0005; β of sex = -0.205, p = 0.001). Linear regression of the putamen-to-caudate SBR ratio with age and sex as independent variables in the healthy controls of the PPMI sample also revealed a significant effect of age and sex (standardized coefficient β of age = 0.216, p =0.002; β of sex = -0.193, p = 0.005). The regression coefficient of age was positive in the latter case, that is, the putamen-to-caudate SBR ratio increased with healthy aging (supplementary Figure S2 below). This suggests that the age-related decline of the SBR is faster for the caudate than for the putamen.

**Impact of the reconstruction method on the effect of age and gender on the putamen SBR**

In order to analyse the impact of the reconstruction method on the effect of age and gender on the putamen SBR, raw projection data from the clinical sample A were reconstructed using the ordered-subset-expectation-maximization (OSEM) algorithm with resolution recovery of the HybridRecon-Neurology tool of the Hermes SMART workstation v1.6 with parameter settings recommended for FP-CIT SPECT by Hermes (effective number of iterations 80, postfiltering with 3-dimensional Gaussian kernel of 7mm full-width-at-half-maximum, uniform attenuation correction with narrow-beam attenuation coefficient 0.146/cm, simulation-based scatter correction, resolution recovery with a Gaussian model). The specific binding ratio (minimum of left and right hemisphere) of the putamen in the OSEM-reconstructed FP-CIT SPECT images (OSEM-SBR) was estimated using hottest voxels analysis as described in the manuscript.

Linear regression of the OSEM-SBR with age and sex as independent variables in the patients with non-neurodegenerative PS of clinical sample A resulted in the following regression line

clinical sample A (non-neurodeg. PS): OSEM-SBR = 2.261 – 0.424*age/100 – 0.140*sex

Mean relative age-related decline in females / males was 2.0% / 2.1%. The effects of age and sex were both statistically significant (standardized coefficient β of age = -0.151, p = 0.038; β of sex = -0.217, p = 0.003).

The total between-subjects variance of the OSEM-SBR in the patients with non-neurodegenerative PS in clinical sample A of 50 years and older explained by the regression model (adjusted R^2^) was 3.9%. For comparison, total between-subjects variance of the OSEM-SBR in all patients of clinical sample A of 50 years and older explained by the group (neurodegenerative PS versus non-neurodegenerative PS) was 61.7%.

The regression line was used to correct the OSEM-SBR for age and sex in all patients of the clinical sample A according to the following formula:

clinical sample A (all patients): corrected OSEM-SBR = uncorrected OSEM-SBR + 0.424*age/100 + 0.140*sex

ROC analyses of uncorrected and corrected OSEM-SBR to identify patients with neurodegenerative PS in clinical sample A showed a small reduction of the area under the ROC curve by age and sex correction that slightly missed statistical significance (area under the ROC curve = 0.949 and 0.943 without and with correction for age and sex, p = 0.059).

These findings suggest that the lack of relevant improvement of the diagnostic accuracy of the putamen SBR by age and sex correction reported in the manuscript is not restricted to a specific reconstruction algorithm but can be generalized to other reconstruction algorithms used in clinical routine.

**Impact of the method used to estimate the SBR on age and gender effects**

A potential impact of the method used to estimate the SBR on age and gender effects was tested in the PPMI sample. For this purpose, the unilateral putamen SBR was also computed by conventional ROI analysis using the anatomical putamen ROIs predefined in MNI space by the Automatic Anatomical Labeling atlas (AAL) [2]. The mean value of the scaled voxel intensity in the AAL ROI was used to calculate the conventional SBR: AAL-SBR = mean scaled voxel intensity in the AAL putamen ROI - 1. In addition, putamen SBR values (PPMI-SBR) provided for download at the PPMI homepage were used (StudyData/Imaging/DaTSCAN/DaTscan_Analysis.csv). The PPMI-SBR estimates have been obtained by automatic placement of small circular ROIs in the putamen [9, 10].

The minimum of left and right hemisphere was used for both AAL-SBR and PPMI-SBR, analogous to the minimum of the hottest voxels putamen SBR of left and right hemisphere that was used in the analyses in the manuscript.

Linear regression of AAL-SBR and PPMI-SBR with age and sex as independent variables in the healthy controls of the PPMI sample resulted in the following regression lines

PPMI sample (HC): AAL-SBR = 1.621 – 0.631*age/100 – 0.168*sex

PPMI sample (HC): PPMI-SBR = 2.130 – 1.116*age/100 – 0.151*sex

Mean relative age-related decline in females / males was 4.4% / 5.0% for the AAL-SBR and 6.2% / 6.8% for the PPMI-SBR. The effects of age and sex were statistically significant for both SBR estimates (AAL-SBR: standardized coefficient β of age = -0.241, p < 0.0005; β of sex = -0.274, p < 0.0005; PPMI-SBR: β of age = -0.235, p = 0.001; β of sex = -0.136, p = 0.045).

The total between-subjects variance explained by the regression model (adjusted R^2^) in the PPMI HC subjects of 50 years and older was 5.8% for the AAL-SBR and 1.5% for the PPMI-SBR. For comparison, total between-subjects variance in all PPMI subjects (HC and PD) of 50 years and older explained by the group (HC or PD) was 71.2% for the AAL-SBR and 75.9% for the PPMI-SBR. Thus, the disease status (PD versus HC) explained 12 (AAL-SBR) to 51 (PPMI-SBR) times more between-subjects variance in the PPMI subjects of 50 years and older than age and sex together.

The regression lines were used to correct AAL-SBR and PPMI-SBR for age and sex in all PPMI subjects according to the following formulas:

PPMI sample (all subjects): corrected AAL-SBR = uncorrected AAL-SBR + 0.631*age/100 + 0.168* sex

PPMI sample (all subjects): corrected PPMI-SBR = uncorrected PPMI-SBR + 1.116*age/100 + 0.151* sex.

ROC analyses of the two putamen SBR estimates to detect the PD patients in the PPMI sample revealed a slight reduction of the area under the ROC curve by age and sex correction. The reduction was statistically significant for the PPMI-SBR (AAL-SBR: area under the ROC curve without / with correction for age and sex 0.991 / 0.989, p = 0.149; PPMI-SBR: area under the ROC curve without / with correction for age and sex 0.996 / 0.993, p = 0.013).

These findings suggest that the lack of relevant improvement of the diagnostic accuracy of the putamen SBR by age and sex correction reported in the manuscript is not restricted to hottest voxels analysis to estimate the putamen SBR but can be generalized to other methods for semi-quantitative analysis used in clinical routine.

**References**

1. Buchert R, Lange C, Spehl TS, Apostolova I, Frings L, Jonsson C, et al. Diagnostic performance of the specific uptake size index for semi-quantitative analysis of I-123-FP-CIT SPECT: harmonized multi-center research setting versus typical clinical single-camera setting. Ejnmmi Research. 2019;9. doi:ARTN 3710.1186/s13550-019-0506-9.

2. Tzourio-Mazoyer N, Landeau B, Papathanassiou D, Crivello F, Etard O, Delcroix N, et al. Automated anatomical labeling of activations in SPM using a macroscopic anatomical parcellation of the MNI MRI single-subject brain. Neuroimage. 2002;15:273-89. doi:10.1006/nimg.2001.0978.

3. Zubal IG, Early M, Yuan O, Jennings D, Marek K, Seibyl JP. Optimized, automated striatal uptake analysis applied to SPECT brain scans of Parkinson's disease patients. J Nucl Med. 2007;48:857-64. doi:10.2967/jnumed.106.037432.

4. Seibyl J, Jennings D, Coffey C, Marek K. Multicenter Evaluation Of Parkinson's Disease Progression Using 123- I I-Ioflupane SPECT: Update From The Parkinson's Progression Marker Initiative Trial. 18th International Congress of Parkinson’s Disease and Movement Disorders Stockholm, Sweden; 2014-06-09.

**Supplementary Figures**

**
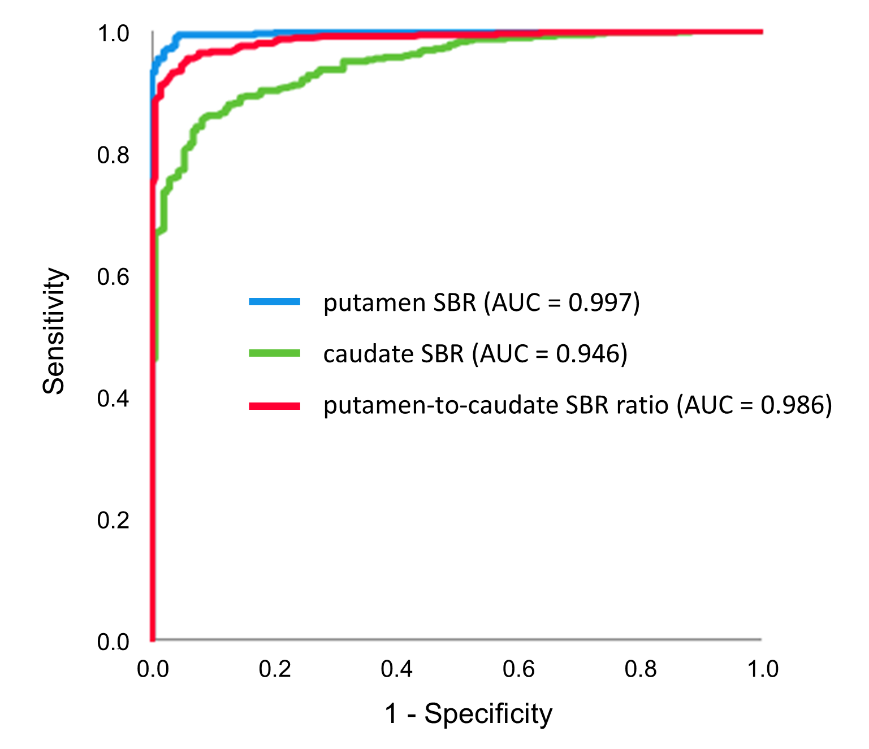
**

**Supplementary Fig. S1** ROC curve of the putamen SBR, the caudate SBR, and the putamen-to-caudate SBR ratio (all without correction for age and sex) for detection of Parkinson’s disease in the PPMI sample. The area under the ROC curves (AUC) is given in the legend.

**
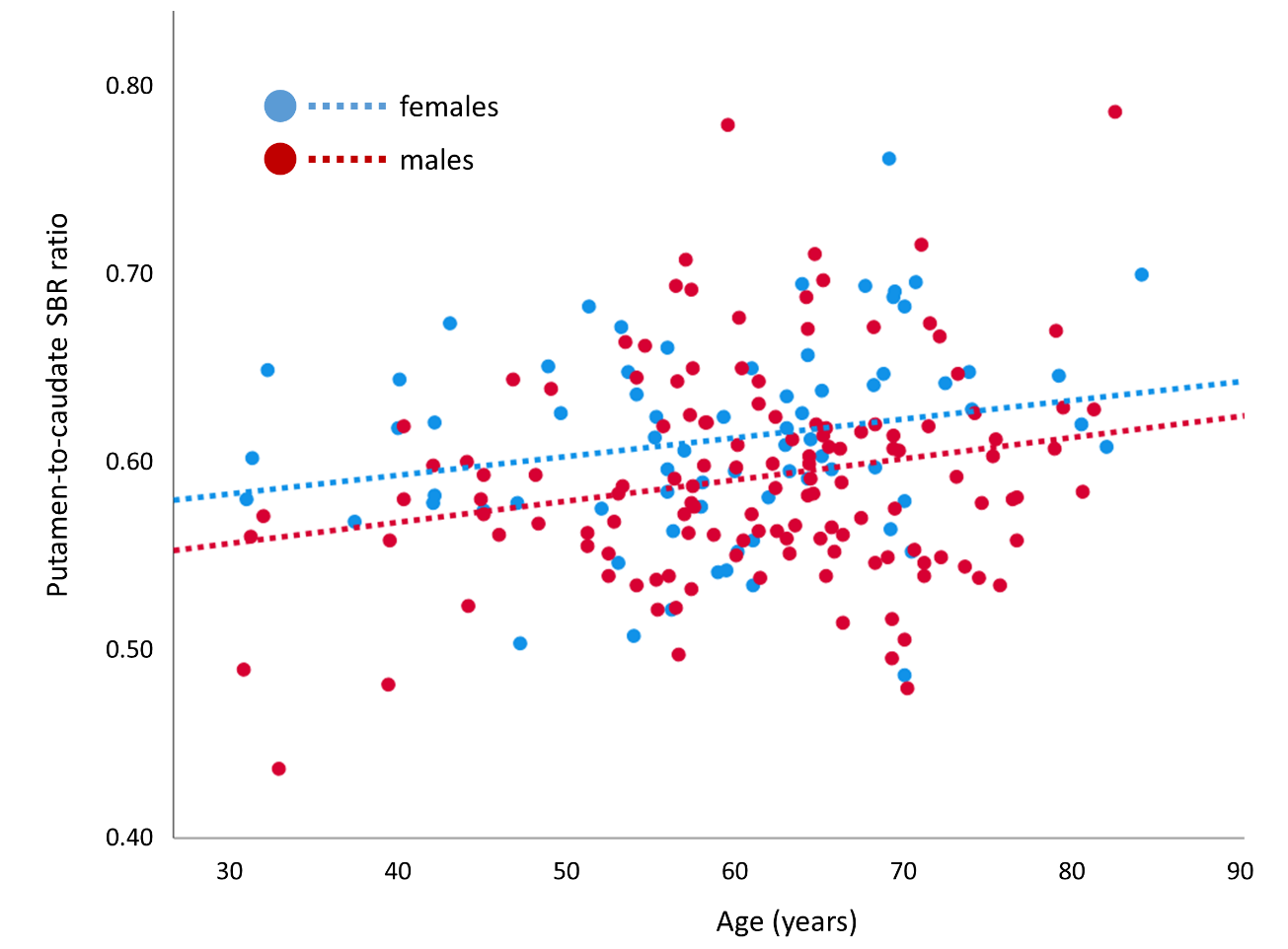
**

**Supplementary Fig. S2** Scatter plot of the putamen-to-caudate SBR ratio versus age in the heathy subjects of the PPMI sample. Sex is indicated by different colors. The dashed lines represent the result of linear regression of the putamen SBR versus age, performed separately for both sexes.
